# Identifying signatures of positive selection in human populations from North Africa

**DOI:** 10.1038/s41598-023-35312-3

**Published:** 2023-05-20

**Authors:** Rocio Caro-Consuegra, Marcel Lucas-Sánchez, David Comas, Elena Bosch

**Affiliations:** 1 Institut de Biologia Evolutiva (UPF-CSIC), Departament de Medicina i Ciències de la Vida, Universitat Pompeu Fabra, Parc de Recerca Biomèdica de Barcelona, 08003 Barcelona, Spain; 2 Centro de Investigación Biomédica en Red de Salud Mental, Instituto de Salud Carlos III, 28029 Madrid, Spain

**Keywords:** Evolution, Genetics

## Abstract

Because of its location, North Africa (NA) has witnessed continuous demographic movements with an impact on the genomes of present-day human populations. Genomic data describe a complex scenario with varying proportions of at least four main ancestry components: Maghrebi, Middle Eastern-, European-, and West-and-East-African-like. However, the footprint of positive selection in NA has not been studied. Here, we compile genome-wide genotyping data from 190 North Africans and individuals from surrounding populations, investigate for signatures of positive selection using allele frequencies and linkage disequilibrium-based methods and infer ancestry proportions to discern adaptive admixture from post-admixture selection events. Our results show private candidate genes for selection in NA involved in insulin processing (*KIF5A*), immune function (*KIF5A*, *IL1RN*, *TLR3*), and haemoglobin phenotypes (*BCL11A*). We also detect signatures of positive selection related to skin pigmentation (*SLC24A5*, *KITLG*), and immunity function (*IL1R1*, *CD44*, *JAK1*) shared with European populations and candidate genes associated with haemoglobin phenotypes (*HPSE2*, *HBE1*, *HBG2*), other immune-related (*DOCK2*) traits, and insulin processing (*GLIS3*) traits shared with West and East African populations. Finally, the *SLC8A1* gene, which codifies for a sodium-calcium exchanger, was the only candidate identified under post-admixture selection in Western NA.

## Introduction

North Africa borders the Atlantic Ocean on the West, the Mediterranean Sea on the North, the Middle East on the Northeast, and the Sahara Desert on the South. Because of its location, the region has witnessed multiple demographic movements that have left recognizable signatures in the genomes of its present-day inhabitants^1–4^. Human presence in the region has been attested since as early as ~ 315 kilo years ago (kya), as evidenced by anatomically modern human fossils found in the Jebel Irhoud site in Morocco^5,6^. Since then, the archaeological record points to a succession of different cultures characterized by different tools and feeding strategies, including the Aterian in the Middle Stone Age^5,7–10^, followed by the Iberomaurusian in the Late Stone Age^11,12^, and the Capsian, who lasted until the arrival of the Neolithic transition^13–17^. These cultures succeed one another in the archaeological record, with important periods of overlap between them. Although the question of genetic continuity or replacement between such cultures is still under debate, genetic continuity at least since Iberomaurusian times is generally accepted^3,18–20^. Just before historical times, an expansion of the Sahara Desert forced population movements towards the Nile Valley in the eastern part of North Africa, eventually giving rise to the first major civilization of the region, the ancient Egyptians (3,000 to 31 BCE). In historical times, the region has seen the arrival of different Mediterranean population groups including, but not limited to, the Carthaginians (ninth-second century [c.] BCE), the Romans (up to the 5th c.), the Vandals (5th c.), the Byzantines (6th c.), the Arabs (7th-16th c.) and the Ottomans (16th c.), as well as those of colonial-period Europeans^21^. Nowadays, North African peoples can be broadly grouped into two major cultural groups: Arabs and Imazighen (sing. Amazigh). The latter, also known by the misnomer *Berber* (from the Latin word *barbarus*, meaning babbling foreigner), are considered the descendants of the Palaeolithic inhabitants of North Africa^22–26^.

Altogether, this complex demographic scenario has left distinct traces in the genomes of present-day North Africans^1–3,27,28^. Furthermore, the genetic diversity observed across the autochthonous populations in the region reflects the multiple episodes of prehistorical and historical unbalanced admixture^2^*.* Henn et al.^1^ pioneered in conducting a genome-wide SNP array-based population genetic analysis of North Africa, showing the presence of at least four main ancestry components: autochthonous (hereinafter, Maghrebi), Middle Eastern-, European-, and West-and-East-African-like (hereinafter, WEA-like). A West-to-East decreasing gradient of the Maghrebi component has been consistently seen in SNP-array and classical marker studies^1,2,29^. Mitochondrial DNA (mtDNA) U6 and M1 haplogroups, identified in present-day North African individuals and ancient mtDNA of human fossils attributed to the Iberomaurusian culture, point to a back-to-Africa migration from the Middle East as its possible source^18,27^. The Middle Eastern-like component shows an East-to-West decreasing gradient^1,2,29^, which according to ancient DNA (aDNA) and genome-wide data, can be mainly traced back to the Late Neolithic gene flow from the Middle East^3,19^, and also to the Arabisation starting in the 7th c.^1,2^. As for the WEA-like component, analysis on SNP-array and mtDNA data are consistent with a continuous gene flow between the 1st c. BCE and the 20th c., probably linked to the trans-Saharan slave trade in Roman, Arab, Ottoman and colonial times^1,2,30,31^. The European-like component can be linked to bidirectional gene flow across the Gibraltar Strait during Neolithic times as evidenced by Y-chromosome^32^ and mtDNA^28,33^ analyses, with other possible contributions of the historical arrival of many Mediterranean peoples and the European-like component present in the Middle-Eastern incomers to the region.

Unfortunately, North Africa has been generally neglected from genetic analyses, and the few population genetics studies performed have been traditionally mostly restricted to mtDNA and Y-chromosome analyses^27–29,32^. Genome-wide data with sufficient geographic representation has only started being collected and analysed in the last decade^1–3,34,35^. However, no attention has been paid to the contribution that positive natural selection has had to the patterns of genetic variation of the inhabitants of North Africa. This is shocking when compared to the large body of research on adaptation focussed on Europe and the remaining African continent. Broadly, consistent adaptive responses to diet, pathogens and UV exposure have been identified in Europe, WEA or both (for an extensive review, see Rees et al.^36^). For example, different SNPs in the *LCT* locus, associated with the phenotype of lactase persistence, have been detected to be under strong positive selection in several European and WEA populations^37^. Moreover, lactase persistence-associated alleles in the Tunisian population show close affinity to South European and Mediterranean groups and thus possibly result from relatively recent gene flow during the settlement of the Roman Empire in NA^38^. Malaria has also been recognized as a strong selective pressure in those geographic areas where the disease is endemic, leading to many adaptive alleles in distinct genes and WEA populations, including *HBA*, *HBB*, *SLC4A1*, *G6PD*, *FY*, *GYPA*, *GYPB*, *GYPC*, *CD36*, and *ICAM1*, among others^39^. Skin pigmentation is another phenotypical trait subjected to strong selection in humans in response to differential UV exposure. Light pigmentation has been hypothesised to have been positively selected to protect against vitamin D deficiency in high latitude areas with low UV radiation, while dark skin pigmentation would be adaptive against folate degradation in high UV radiation areas^40^. Accordingly, distinct adaptive variants are recognized in pigmentation genes such as *SLC45A2* and *TYRP1* in Eurasian populations; *HERC2*, *DDB1,* and *MFSD12* in WEA populations; and *SLC24A5* and *OCA2* in populations from both geographical areas^40^. One of the challenges to analysing local genetic adaptation in North Africa is the complexity of the admixture patterns of the autochthonous populations with several historical external sources, which might complicate the identification of true adaptive signals. Notwithstanding, admixture events can also introduce beneficial mutations in the recipient populations in a process known as adaptive admixture (discussed in Cuadros-Espinoza et al.^41^).

In this work, we compile publicly available genome-wide genotyping data from a total of 190 North African individuals and investigate for signatures of positive selection using statistical tests based on allele frequencies and linkage disequilibrium. We also interrogate the genomes of different source populations that are descendants of those that putatively admixed with North Africans, and combine these analyses with the inference of ancestry proportions in the North African individuals to explore whether the adaptive signals identified are geographically restricted to North Africa, shared with external sources of admixture, and thus probably result from positive selection already occurring in the source population before the admixture event, or whether they result from post-admixture selection on external ancestry components.

## Results

### Population structure

**Figure 1:**
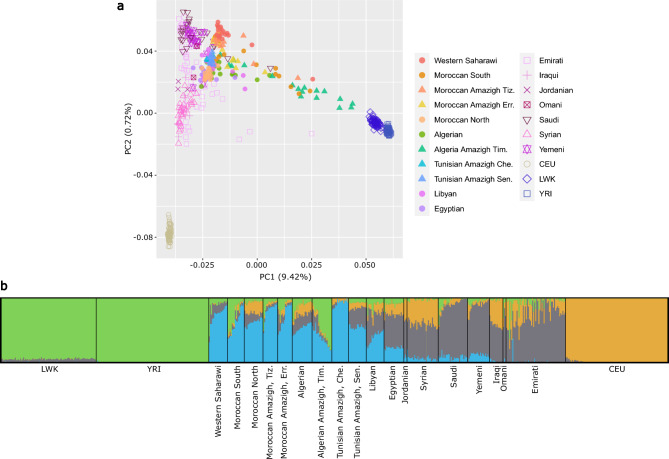
Population structure analysis of the North African dataset. (**a**) PCA of North African individuals (full coloured symbols) and reference populations (empty symbols) from West and East Africa (YRI and LWK, respectively), Europe (CEU), and the Middle East. (**b**) ADMIXTURE analysis at K = 4 with the same samples. YRI, Yoruba from Ibadan (Nigeria); LWK, Luhya in Webuye (Kenya); CEU, Utah residents with North- and West- European ancestry.

Population structure was assessed analysing 639 individuals from 22 populations (including 190 North Africans, see Table 1 and “Materials and Methods” section). After pruning, we performed global population structure analyses including principal component analysis (PCA) and ADMIXTURE. In the PCA (Fig. 1a), the first principal component (PC1) shows a Europe to West- and East-Africa (WEA) cline, clearly separating individuals according to their proportions of WEA-like ancestry. On the other hand, PC2 separates European populations from the rest. North African (NA) and Middle Eastern (ME) samples form two dispersed but overlapping clusters accounting for the varying proportions of admixture with WEA-like, European-like, and ME-like ancestries. When comparing NA and ME to the other samples, NA appears closer to the WEA cluster than ME does, with Algerian Imazighen being the closest non-WEA population to WEA samples. In PC3 (Supplementary Fig. S1), we can further distinguish NA and ME samples, as they form a West-to-East clinal pattern, except for the Tunisian Imazighen from Chenini.

**Table 1:** Populations compiled in this study and population groups used in the selection analyses.

Populations and population groups	Sample size	Ref.
North African (NA)		
Western North African (NAW)		
Algerian Amazigh, Timimoun	19	^2^
Algerian, Alger	19	^1^
Moroccan Amazigh, Errachidia	14	^2^
Moroccan Amazigh, Tiznit	14	^2^
Moroccan North	18	^1^
Moroccan South	16	^1^
Western Saharawi	18	^1^
Tunisian Amazigh, Sened*	17	^2^
Tunisian Amazigh, Chenini*	16	^1^
Eastern North African (NAE)		
Egyptian	19	^1^
Libyan	17	^1^
West and East African (WEA)		
Yoruba in Ibadan, Nigeria (YRI)	108	^96^
Luhya in Webuye, Kenya (LWK)	91	^96^
European		
Utah residents with North- and West- European ancestry (CEU)	98	^96^
Middle Eastern (ME)		
Iraqi	13	^98^
Jordanian	3	^98^
Omani	3	^98^
Saudi	28	^98^
Syrian	11	^98^
Syrian	19	^2^
Emirati	57	^98^
Yemeni	21	^98^
TOTAL	639	

Each row contains a population, its sample size, and the reference from which it was obtained. For the population structure analysis, we considered each population unit separately. For the selection analyses, populations were grouped to increase sample size. All North African (NA) genotypes were considered as a unit to compute F_ST_ and XP-EHH between NA and any external source, while for the iHS and LAD tests, NA populations were divided into Western and Eastern NA (NAW and NAE, respectively). Selection analyses were similarly conducted on the West- and East- African (WEA) group and the European group, whereas the Middle Eastern (ME) group was only used in those selection tests based on population structure (Ohana and LAD). *Tunisian Imazighen individuals were excluded from all analyses of positive selection except for Ohana.

Consistent with the PCA results, the ADMIXTURE analysis shows high heterogeneity in the ancestry proportions identified within and between NA populations. In K = 4 (lowest cross-validation [CV] error), a possibly Maghrebi component is present in all NA samples while almost absent elsewhere (Fig. 1b). The predominant component in WEA samples is detected in varying proportions throughout NA individuals, with high proportions in Algerian Imazighen and some Southern Moroccan samples. The major ancestry component identified in the European samples also shows differential but generally minor proportions across NA populations, being almost absent in the Amazigh groups, Southern Moroccans, and Western Saharawi. A putatively autochthonous ME component (predominant in ME samples) is similarly present in most NA populations with proportions that reproduce the East-to-West decreasing gradient seen in PC3 of the PCA and that contrast the West-to-East opposite decreasing gradient of the Maghrebi component. The Tunisian Amazigh population from Chenini is an exception to such clinal patterns, with almost negligible proportions of non-NA components. In fact, in K = 6, within the range of lowest CV errors and showing convergent results in the ten computed runs, this population exhibits a practically private component at very high proportions (Supplementary Fig. S2). This is compatible with genetic isolation after a relatively recent bottleneck, followed by strong genetic drift and small effective population sizes, as supported by Ne, ROH and IBD analyses (see Supplementary Methods, Supplementary Figs. S3 and S4 and Supplementary Table 1). Moreover, a similar pattern is observed for Tunisian Amazigh individuals from Sened. To avoid potential biases caused by the particular demographic history of the two Tunisian Imazighen populations sampled, we have excluded both populations from all analyses of positive selection (except for Ohana).

### Analysis of positive selection

To identify candidate genes for selection in NA populations, we used multiple statistical tests and population comparisons to ensure the detection of positive selection under different scenarios. In particular, we (i) combined F_ST_ and cross-population Extended Haplotype Homozygosity (XP-EHH) analyses to identify extremely differentiated regions of the genome with unusually long-range linkage disequilibrium when comparing NA with either CEU or WEA (Supplementary Tables S2–3); (ii) computed the integrated Haplotype Score (iHS) grouping the NA populations from the West (NAW; i.e., Western Sahara, Morocco, and Algeria) and those of the East (NAE; i.e., Egypt and Libya) to identify contrasting extensions of haplotype homozygosity between the chromosomes carrying the ancestral and the derived allele of a given polymorphism within these groupings (Supplementary Tables S4–5); (iii) run Ohana, which uses population structure information to identify genomic regions whose allele frequencies cannot be explained solely by the ancestry components as inferred genome-wide, to specifically identify candidate SNPs under selection in the NA ancestry component (Supplementary Table 6); and (iv) look for local ancestry deviations (LAD) in the genomes of the NAW and NAE populations to identify loci with significant deviations in their local ancestry proportions (Supplementary Table S7). The F_ST_, XP-EHH and iHS statistics were also computed on European and WEA populations, considered here as putative sources for the different external ancestry components detected in present-day NA genotypes. Subsequently, matches between the obtained candidate regions across populations in these main geographical regions and selection tests were checked and further intersected with selection signals reported in Southern European populations (Supplementary Tables S8–11).

#### North African-specific signals

Signals of positive selection exclusively found in NA populations include genes associated with insulin processing, such as *KIF5A,* identified within the top 0.1% highest scoring SNPs for iHS in NAE (Supplementary Table S5), and several candidates related to the immune system, such as *IL1RN* and *TLR3*, detected within the top 1% of the F_ST_ and XP-EHH combined *p*-values in NA when compared to Europe (Supplementary Table S3). The *KIF5A* gene encodes a kinesin involved in intracellular organelle transport, including insulin-charged secretory vesicles and vesicular transport of proteins in neurons, by being part of a multi-subunit complex that functions as a microtubule motor^42^. However, it has also been described to play a role in the adaptive immune system by transporting antigen-loaded MHC class II molecules^43^. *IL1RN* encodes a member of the interleukin 1 receptor antagonist that modulates related inflammatory responses, while *TLR3*, codifies a Toll-like receptor protein involved in pathogen recognition and anti-viral inflammatory responses of the innate immune system^44^. Moreover, both genes have been associated with changes in susceptibility to infection with multiple pathogens including Ebola^45^, COVID-19^46^, papillomavirus^47^, *Helicobacter pylori*^48^, herpes simplex, varicella-zoster^49^, and HIV-1^50^. *BCL11A*, a gene associated with various haemoglobin-related traits, was also found within the top 1% F_ST_—XP-EHH signals in the NA vs Europe comparison (Supplementary Table S2). Notably, *BCL11A* has been suggested as a therapeutic target for the treatment of β-thalassemia and sickle cell anemia^51^, and common variants in the gene are associated with the persistence of foetal haemoglobin (HbF) into adulthood, as well as with milder presentations of both diseases^51^. Finally, some of the signals identified as unique to NA populations in our analyses (when compared to CEU and WEA) comprise candidate genes that have been previously identified under positive selection in Southern European populations (Supplementary Table S11) and thus may probably relate to common selective pressures across the Mediterranean region. Among those, we found *BNC2* identified with the iHS in NAE (Supplementary Table S5) as well as in Toscani and two North Eastern Italian populations^52^, which has been associated with facial pigmentation^53^; and *PTPRD* which we detected in NA when compared to CEU (with F_ST_ and XP-EHH; Supplementary Table S2) and has been previously described under positive selection in all the Italian populations explored by Cocca et al.^52^, although with signals on different markers, as well as in several malaria endemic regions of Asia^54^.

**Figure 2:**
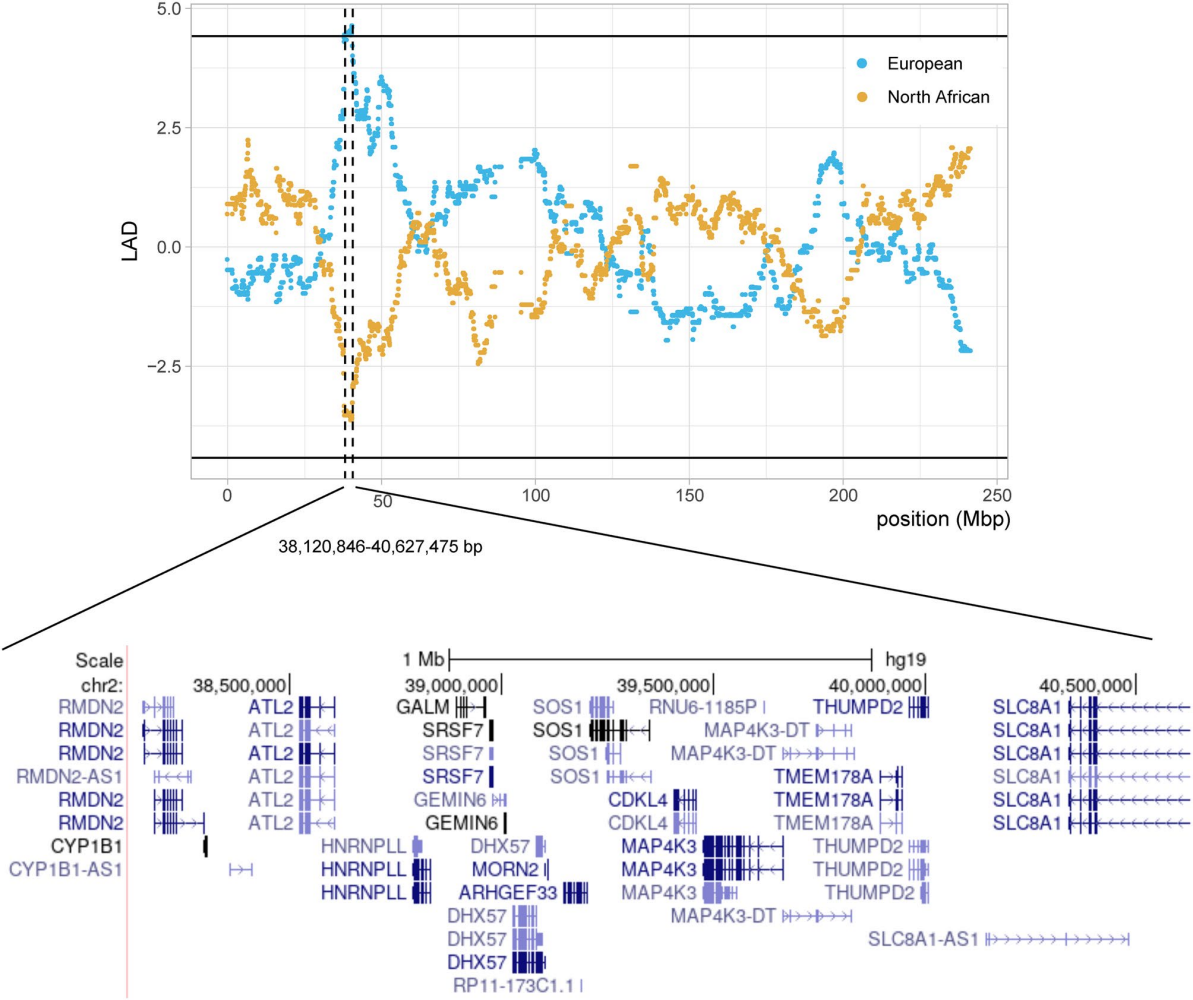
Local Ancestry Deviation (LAD) identified in chromosome 2 of Western North African populations (NAW). The horizontal lines mark the threshold of |LAD| above 4.42, indicative of putative post-admixture selection^120^. A zoom in of the genes contained within the genomic region surpassing the defined threshold is also represented as extracted from the UCSC Genome Browser on Human (GRCh37/hg19).

Our LAD analysis detected a large region in chromosome 2 (38,120,846–40,627,475 bp; hg19) in the NAW group with a significant deviation of European-like ancestry, reaching up to 33.1% of the ancestry component while the average of European-like component estimated genome-wide in NAW is 15.3% (Fig. 2, Supplementary Table S7). After exploring those SNPs displaying the highest allele frequency differences between Europe and WEA across the whole region and filtering for variants with a CADD PHRED score > 10 (see Supplementary Table S7), we only identified the intronic SNP rs741286 within the *SLC8A1* gene, which has been associated with salt-sensitive hypertension as well as electrocardiographic traits^55,56^. Interestingly, *SLC8A1* has also been identified as candidate for positive selection with the iHS in several populations across Italy^52^ (Supplementary Tables S10–S11).

#### Shared signals with Europe

Among those candidate genes for positive selection shared between NA and Europe (see Supplementary Table S9), we identified multiple genes related to the immune system including *IL1R1* and *CD44*, detected within the top 0.1% of iHS for NAE; and *JAK1* identified within the top 0.1% iHS signals in NAE and with a significant log-likelihood ratio (LLRT > 15) in Ohana (Supplementary Tables S5 and S6). The three immune system-related genes (*IL1R1*, *CD44* and *JAK1*) are part of the “cytokine signalling in the immune system” Reactome pathway. Whereas *IL1R1* is an interleukin-1 receptor, *CD44* encodes a cell-surface receptor involved in inflammation and response to bacterial infection^57^, and *JAK1* encodes a tyrosine kinase involved in both interferon and interleukin signal transduction (O’Shea et al.^58^ and references therein). Previous studies had already identified *JAK1* under selection in West Eurasian populations^59^.

**Figure 3:**
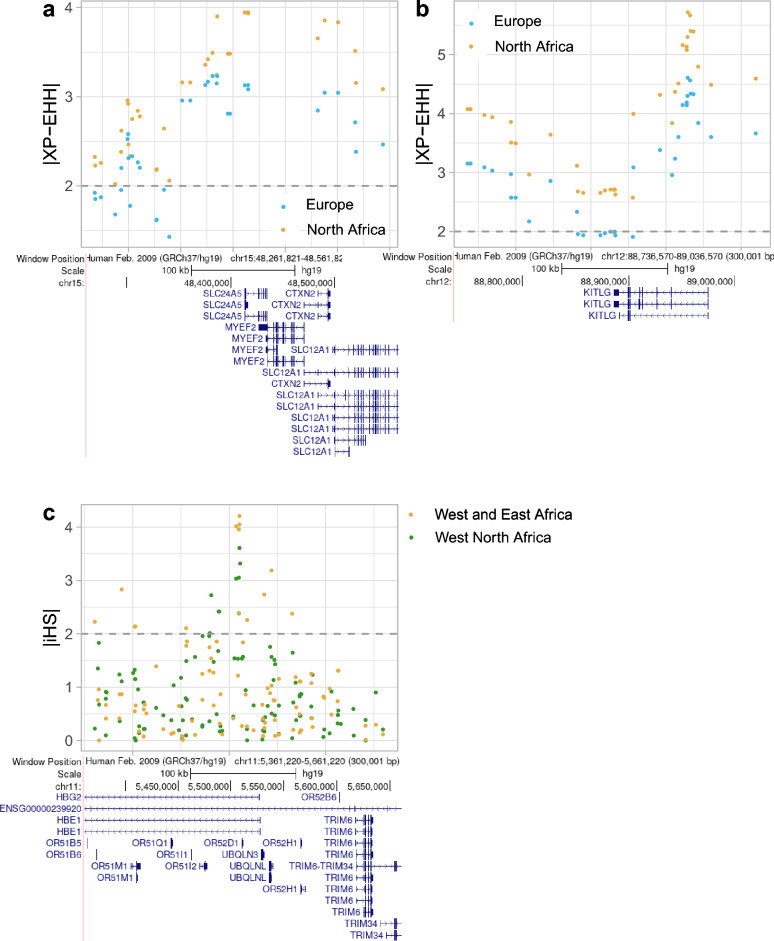
Signals of positive selection identified in North African (NA) populations that are shared with European or West and East African (WEA) populations. (**a**) |XP-EHH| scores for the NA and European populations (when compared against WEA) in chr15:48,261,821–48,561,821, which contains the *SLC24A5* gene. (**b**) |XP-EHH| scores for the NA and European populations (when compared against WEA) in chr12:88,736,570–89,036,570, which comprises the *KITLG* gene. (**c**) |iHS| scores for Western NA and WEA in chr11:5,361,220–5,661,220, where the *HBE1* and *HBG2* genes reside. The horizontal dashed lines represent the minimum threshold of significance for each statistic. Within each genomic region, gene tracks were extracted from the UCSC Genome Browser on Human (GRCh37/hg19).

Other shared signals between NA and Europe include two well-known candidate loci for selection recognized to contribute to skin pigmentation lightening in Europeans. These are *SLC24A5*, detected here in the joint F_ST_ and XP-EHH analysis comparing NA vs WEA (Fig. 3a; Supplementary Table S3); and *KITLG*, identified in the joint F_ST_ and XP-EHH analysis comparing NA vs WEA (Fig. 3b; Supplementary Table S3), as well as with the iHS statistic in NAE (Supplementary Table S5). The *SLC24A5* locus has been extensively reported to be under positive selection in West Eurasia^59,60^, with the A allele of the rs1426654 SNP being fixed in West Eurasian populations and experimentally proven to cause lighter skin pigmentation^61^. Although this SNP is missing in our filtered dataset, three other SNPs in the *SLC24A5* locus show a strong allele frequency differentiation when comparing NA to WEA populations (rs2250072-A 0.78 vs 0.08; rs2675346-C 0.93 vs 0.33; rs2433354-C 0.92 vs 0.34). Of those, rs2250072 is in moderate linkage disequilibrium (LD) with rs1426654 (r^2^ = 0.534 and D’ = 0.94 in WEA). Similarly, the C allele at rs12821256 in the regulatory region of the *KITLG* gene has been associated with blond hair^62,63^ and identified under positive selection in Eurasian populations (~ 12% freq.)^59,64–66^. Although we detected up to ten SNPs with significant statistic selection scores around *KITLG* displaying contrasting allele frequencies between NA and WEA populations (Supplementary Table S3), none of them is in LD with the above-reported variant.

#### Shared signals with WEA

Among the candidate regions for positive selection identified in NA and WEA populations (see Supplementary Table S10), we detect multiple genes associated with haemoglobin-related traits. These include *HPSE2* within the top 0.1% iHS signals in NAE (Supplementary Table S5), as well as the *HBE1* and *HBG2* genes within the top 0.1% iHS values in NAW (Fig. 3c; Supplementary Table S4). Interestingly, distinct genetic variants in these three genes are associated with increased production of foetal haemoglobin (HbF) (reviewed in Menzel et al.^67^) and *HBE1* and *HPSE2* had also been previously detected as shared candidate genes for positive selection in a number of Italian populations^52^ (Supplementary Tables S10–S11). Despite the specific outlier SNPs for signals of selection identified here not being annotated elsewhere, two of them (rs394893 and rs394620 in *HBE1* and *HBG2*, respectively) are in moderate LD with rs372091 (r^2^ ~ 0.57; D’ ~ 0.78 in WEA), associated with resistance against severe malaria^68^. Similarly, we detected *CSMD1*, which has been associated with severe malarial anaemia^69^, as a shared candidate for positive selection in NA and WEA populations (detected within the top 0.1% iHS signals in NAE and WEA, and within the top 1% F_ST_-XP-EHH values when comparing NA to CEU; Supplementary Tables S2 and S5) as well as in all the Italian populations analysed by Cocca et al.^52^ (Supplementary Tables S10–S11). Additionally, *GLIS3*, a gene associated with glucose metabolism, and *DOCK2*, related to the immune system, are both detected within the top 0.1% iHS values in NAE (Supplementary Table S5) but also identified in WEA. Notably, *GLIS3* encodes a zinc finger protein involved in pancreatic β-cell development, and multiple variants along the gene have been extensively associated with diabetes mellitus (reviewed in Scoville et al.^70^). *DOCK2* is involved in lymphocyte migration as a response to chemokines and is identified as part of the “Host interactions of HIV factors” Reactome pathway^71^.

## Discussion

In this study, we performed the first analysis of positive selection in North Africa. To do so, genotyping data were collected for 190 individuals from eleven populations across North Africa (NA), together with that of Middle Eastern (ME), West and East African (WEA), and European (CEU) populations from various sources. The results of PCA and ADMIXTURE analysis show that present-day NA populations are genetically highly heterogeneous. Their predominant ancestry components are an autochthonous Maghrebi component, decreasing in a West-to-East gradient, and a ME-like component, decreasing in the opposite direction (East-to-West). In lower amounts, we also observe varying proportions of WEA-like (highly present in Algerian Imazighen) and European-like ancestry components. This pattern of admixture is consistent with previous genome-wide array analyses^1,2^, in which the NA ancestry picture was depicted as an amalgam of the four aforementioned components. The Tunisian Imazighen populations from Chenini and, to a lesser extent, from Sened, are an exception to the clinal patterns observed in most of the other NA populations in our dataset. For instance, in the ADMIXTURE analysis, the Chenini population barely shows any components other than the Maghrebi, and in larger Ks, it presents a private ancestry component. This can be the result of a combination of strong genetic drift and a small effective population size after a recent bottleneck in the population. Such a pattern has been previously reported in the same population using whole-exome sequencing data^35^ and depicts the large demographic heterogeneity in NA.

Candidate genes under positive selection identified exclusively in NA populations should correspond to adaptive signals specific to the NA region, resulting from either selection events in the Maghrebi component, or post-admixture selection when implying external components with LAD. The LAD analysis did not reproduce any of the shared candidate genes for positive selection identified with the remaining tests. Notwithstanding, we did detect a large region in chromosome 2 of NAW individuals exhibiting a significant excess of European-like ancestry. Even if alternative causal genes within the region cannot be discarded, *SLC8A1* was one of the genes presenting higher allele frequency differentiation between European and WEA populations across the region and had been previously identified under positive selection in several Italian populations^52^. This pattern is compatible with a scenario of post-admixture selection, where the European-like component would provide a selective advantage to NAW populations. Notably, this chromosomal region was also identified within the top 0.1% iHS values in WEA, with the highest-scoring SNPs showing contrasting allele frequency trends and higher homozygosity values on the non-favoured alleles found in NAW populations. *SLC8A1* encodes a sodium/calcium exchanger highly expressed in the heart, where it plays a role in returning cardiac muscle to a resting state^72^. Its association with salt-sensitivity hypertension has been experimentally demonstrated^56^, and multiple *SLC8A1* variants have been associated with electrocardiographic traits and salt-sensitivity hypertension^55,73,74^. Interestingly, other hypertension-associated genes have been identified under positive selection in WEA populations^75–77^, where hypertension is highly prevalent^78^. Furthermore, susceptibility variants for hypertension have been shown to follow a latitude clinal pattern, with decreasing hypertension susceptibility towards colder areas, supporting the hypothesis that salt retention might be adaptive in populations living in hot, humid areas with low dietary salt availability, and that selection for differential susceptibility occurred during the Out of Africa expansion when humans expanded to colder environments^75–77^. Therefore, our results could suggest that the post-admixture selection signal detected in NAW is probably related to adaptation to colder environments.

Shared signals with either Europe or WEA and NA probably indicate selection events occurring in the source population that after admixing with NA populations, might (i.e., adaptive admixture, an expected scenario in case of shared selection pressures across geographical regions) or might not have remained adaptive in NA (i.e., a spurious selection signal from the external ancestry component)^41^. For instance, the identification of *SLC24A5* and *KITLG* as candidate genes for positive selection in both NA and European populations could easily result from a shared selective pressure related to the lower radiation levels of their corresponding locations when compared to that of the ancestral lower latitudes. Light skin pigmentation is likely an adaptive mechanism facilitating vitamin D biosynthesis, important for immunity and calcium homeostasis^79,80^. The link between some variants in *SLC24A5* and *KITLG* under selection in West Eurasian populations and lighter skin pigmentation is well established^59–66^. Although the allele frequency differentiation between NA and WEA around these genes is strong enough to point to positive selection, we cannot fully discard the possibility of detecting a residual signal, despite the proportion of the European-like component in NA being relatively low. Similarly, *CSMD1* and the *HBE1, HBG2* and *HPSE2* genes were identified as candidates for positive selection in both NA and WEA populations, probably because of the historical presence of malaria in both Sub-Saharan Africa and the Mediterranean region as a common selective pressure. Multiple variants within the *HBE1, HBG2* and *HPSE2* genes have been associated with increased production and/or persistence of HbF into adulthood^67^ and, as a consequence, with a milder presentation of β-globin diseases including β-thalassemia and sickle cell disease^51^. Variants in *BCL11A*, a candidate gene of selection identified exclusively in NA populations, have also been associated with the persistence of HbF^51^. Although in Andean and Tibetan highland populations, *HBE1* and *HBG2* have been reported as candidates for selection, probably because of their impact on oxygen transportation^81,82^, to our knowledge, this is the first study where such genes are detected as candidates of positive selection in NA and WEA populations. The long-term selective pressure that malaria has exerted over human populations inhabiting areas where the disease is endemic has favoured the presence of several genetic variants that provide resistance to malaria at relatively high frequencies. However, such genetic adaptations often result in an increased prevalence of several β-globin diseases^67,83,84^. Here, we identified *BCL11A* in NA and *HPSE2*, *HBE1* and *HBG2* in both NA and WEA populations as new candidate genes for positive selection, which we hypothesize could probably protect against the most severe presentations of the β-globin diseases commonly found in the geographical areas where malaria is endemic. While signatures of positive selection had been reported in Southern Europe for the *CSMD1*, *HBE1* and *HPSE2* genes^52^, other putative targets of adaptation related to response to *Plasmodium falciparum* previously described in Sardinians, such as the *CR1*^85^ and *THBS1* genes^86^ were not found under the top selection signals identified here in NA populations.

Finally, the Major Histocompatibility Complex (MHC), a genomic region containing several genes involved in both the adaptive and the innate immune responses is consistently identified within the top iHS-scoring regions in all three groupings of NA, European, and WEA populations. Because of the hypervariability that characterises the region, we could argue that balancing selection, and not positive (or directional) selection, would be acting to maintain such variability. This argument is widely accepted and documented in multiple reviews^87–89^. Indeed, signatures of increased linkage disequilibrium around a target of recent balancing selection can easily be confused with signals of recent positive selection^90^. Although the analysis of the full site frequency spectrum could be used to test whether a pattern of increased diversity and excess of common polymorphism as expected under balancing selection is confirmed in NA for the MHC region, sequencing data is not yet available for these populations. Moreover, pathogen diversity is not only reported as the main driving force behind the high genetic variability in the HLA genes^91,92^, but also elsewhere^89,93,94^. Taken together, our results are consistent with pathogens constituting the main driver of natural selection in humans, as the strongest candidate genes under selection identified are those related to the immune system.

Even though an increase in the efforts for collecting and generating genome-wide datasets of underrepresented populations has been made, to date, the availability of genomic data from North African populations is still scarce. When considering the vast genetic heterogeneity reported both between and within populations in the area, the problem is exacerbated. Because of the limited sample size available for each geographical location, we analysed signatures of positive selection mostly grouping the whole dataset of NA populations. Although this strategy increases the statistical power of our analysis, it could also dilute the effect of differential selective pressures acting on a specific geographical area. To try to tackle this, in the iHS and LAD analyses, we examined the NA populations from the West separately from those of the East. Ideally, though, individuals should be grouped based on common selective pressures, and considering common demographic and cultural backgrounds. Other limitations of our study are the ascertainment bias introduced when using SNP array data, as well as the subjacent lower power to identify the true causal variant under adaptation. This scenario often leads to an additional bias towards reporting previously recognized candidate variants or genes, and against those for which no information is available. In the context of detecting positive selection in admixed populations, the reliability of the proxies chosen as source populations should also be carefully examined. The recent action of positive selection in the present-day populations that have been used as proxies could also lead to spurious results, since the allele frequency spectrum and haplotype patterns of the genomic regions targeted by positive selection that are shared with the admixed population, would be altered in both, mimicking an adaptive admixture scenario^41^. Alternatively, when a sufficiently strong selection signal identified in the proxy (and source) is also detected in the admixed population, discerning between a case of adaptive admixture or a residual signal is not trivial, especially in recent admixture scenarios^41^. Moreover, because we are using an outlier approach where genome-wide cut-offs are defined rather arbitrarily, candidate genes identified only in NA might not be exclusive when a different cut-off is chosen. Recently, methods with the ability to model complex demographic scenarios have been developed (e.g. Relate^95^), and some of these are even capable of inferring allele frequency trajectories over time (e.g. CLUES^66^). Together, these novel strategies would help to disentangle the different scenarios facilitating local genetic adaptation in North Africa, but they require the use of sequencing data.

In conclusion, in this first study of positive natural selection performed in North African populations, we were able to identify several candidate genomic regions for positive selection and characterise whether these adaptive signatures were private or shared with European and WEA populations. However, with the limitation of the current genomic data available and methods used we could not distinguish between a scenario of positive selection acting in the source populations only and that of adaptive admixture when encountering shared signals of selection. Thus, caution should be taken when interpreting shared signals of positive selection between NA and the European and WEA populations used as source populations for admixture. The generation of sequencing data from a variety of geographic locations within North Africa is an essential future step required for further understanding of the nature of such adaptive signals.

## Materials and methods

### Samples and genotypes

We used Affymetrix 6.0 array data from 190 NA individual samples and 19 Syrian (ME) samples, retrieved from the previous work of Arauna et al.^2^. These data are a combination of newly genotyped data in that work^2^ with data already published by Henn et al.^1^. Upon retrieval, the dataset had already been filtered by missingness per individual (> 0.1), relatedness based on Identity by State (IBS > 0.85), missingness per SNP for each population (> 0.1) and by Hardy–Weinberg equilibrium (HWE at *p* < 0.05) for each population, resulting in a dataset of 486,252 SNPs.

We next merged the dataset with sequencing data from 98 CEU, and from 91 LWK, and 108 YRI individuals from the 1KGP^96^ to be used as proxies for the European, and WEA ancestries, respectively. Before merging the datasets, we used VCFtools 0.1.14^97^ for QC. We excluded non-biallelic sites and indels; we applied a ≤ 3rd-degree relatedness filter (–relatedness2) resulting in the exclusion of the genotypes of 1 CEU, 8 LWK, 1 Algerian Amazigh, and 2 Tunisian Amazigh individuals from Chenini; and checked that missingness per site (–max-missing) and individual (–missing-indv) did not exceed the 10%. At this point, the dataset contained 404,336 sites and 503 individuals from 15 populations.

We further completed the compiled dataset with sequencing data generated on ME individuals by Almarri et al.^98^. Individual sample files were merged using BCFtools 1.9^99^ with the merge option -0, which sets missing genotypes to reference/reference. For compatibility with the rest of the data, we used CrossMap^100^ to convert the coordinates from the GRCh38 to the GRCh37 human genome reference assembly. We removed 99 sites with duplicate IDs using PLINK 1.9^101^. After merging the datasets with BCFtools and restricting data to common sites, we used VCFtools for QC. We removed non-biallelic sites and indels, as well as one related Syrian individual. 24,558 sites did not pass the missingness < 10% filter. Additional filters resulted in removing 38 sites failing the per population Hardy–Weinberg test at 10e-8 significance, and 66,604 sites with a minor allele frequency (MAF) < 0.05. Finally, we kept only those sites compressed in the 1KGP strict accessibility mask, applied using BCFtools. The final dataset contained 376,638 sites and 639 individuals from 22 populations (Table 1).

### Population structure analysis

We performed a PCA using the SmartPCA tool included in the EIGENSTRAT stratification correction method implemented in the EIGENSOFT software package 6.0.1^102^. Data was pruned for LD using PLINK 2.0 with sliding windows of 50 kb, a step size of 5 SNPs and a square correlation coefficient (r2) threshold of 0.5, keeping 224,808 sites.

We explored ancestry patterns in the pruned dataset with ADMIXTURE 1.3^103^ applied in unsupervised mode and with a range of K = 2 to K = 10 ancestral clusters. We computed 50 independent runs for each K using a random seed in each run. CV errors were assessed at each run and mean values were calculated to learn the range with minimum error. Common modes among different runs for each K were identified with pong in greedy mode^104^, which was also used for visualization and plotting of the results.

### Positive selection analysis

To identify signals of adaptation in NA, we first used VCFtools to compute F_ST_^105^ against WEA and Europe, separately. Then, we used Selscan 1.2.0^106^ to calculate and normalise the XP-EHH^60^ test against the same populations as for F_ST_, after phasing the dataset with SHAPEIT 4.1.3^107^. We extracted the top 1% scoring SNPs from both population comparison-based tests (F_ST_ and XP-EHH) and combined the rank-based *p*-values of those in common using Fisher’s combined score (F_CS_), as in Deschamps et al.^108^. The resulting set of outlier SNPs was annotated using VEP^109^ from Ensembl.

In addition, we performed a population-specific haplotype-based test, the iHS^110^, using Selscan on phased data previously polarised based on the ancestral allele information obtained from the 1KGP dataset. Normalisation was applied by MAF bins. We computed iHS for the West and East NA samples, separately (NAW: Western Sahara, Morocco, and Algeria; and NAE: Libya and Egypt; respectively), as well as for WEA and Europe. The 0.1% top-scoring SNPs were extracted and annotated with VEP.

Next, we applied Ohana, a maximum likelihood method that incorporates admixture information to identify signals of selection in specific ancestral components^111^. We followed the described pipeline and applied the test assuming four ancestral components. For each component, we extracted the SNPs with log-likelihood ratios (LLRT) larger than 15 and annotated them with VEP.

The complete list of candidate genes for positive selection was further annotated using GeneCards^112^ and interrogated with data from the GWAS Catalog^113^, Gene Ontology (GO)^114^, OMIM^115^, Reactome^116^ and KEGG^117^. Then, we checked for matches among genes identified by the different tests performed in NA (or NAW/NAE) and proxy populations used as sources. Candidate genes for positive selection identified in both NA and proxies indicate selection at least in the proxy population and possibly adaptive admixture.

Finally, we used RFMix v2.03^118^ to infer local ancestry and identify LAD that could be attributed to post-admixture positive selection. As proxies for reference populations, we used 20 CEU samples, and 10 YRI plus 10 LWK samples to represent the European and WEA components, respectively. To select a set of reference samples for NA and ME populations, we used global ancestry proportions obtained for K = 4 (lowest CV error) in the ADMIXTURE analysis. After excluding Tunisian Imazighen individuals because of their high degree of genetic isolation, we extracted 22 NA samples and 18 ME samples showing NA and ME ancestral components greater than 85% and 90%, respectively. These percentages of global ancestry were chosen based on a compromise between the maximum ancestry and sufficient sample size. Because we used proxies as reference populations and these proxies are admixed, we used the—reanalyze-reference option and 5 expectation–maximization (EM) iterations. Finally, we processed the results using a modified version of Alicia Martin’s pipeline^119^, and computed the local ancestry proportions per SNP and population group, separating NAW and NAE. Regions with significant LAD (> 4.42 as seen in Bhatia et al.^120^) were extracted for each ancestry and annotated using VEP. The obtained list of candidate genes for selection was further annotated and interrogated as described before.

### Ethical approval

The study was approved by the institutional review board CEIm – Parc de Salut MAR (reference number 2019/8916/I).

## Supplementary Information

Supplementary Information 1.

Supplementary Information 2.

Supplementary Information 3.

Supplementary Information 4.

Supplementary Information 5.

Supplementary Information 6.

Supplementary Information 7.

## Data Availability

This study is based on previously published genomic data from diverse sources (see details in Table 1). Data from North African individuals were collected from Henn et al.^1^ and Arauna et al.^2^. Data from Middle Eastern individuals were collected from Almarri et al.^98^. The rest of the data were collected from the 1000 Genomes Project dataset^96^.
