# Supplementary material for: Identifying signatures of positive selection in human populations from North Africa
Source: Sci Rep. 2023 May 20;13:8166. doi: 10.1038/s41598-023-35312-3 (PMC10199912; doi:10.1038/s41598-023-35312-3)
Supplement: Supplementary file 1 — Supplementary Information 1. [file 41598_2023_35312_MOESM1_ESM.pdf]

Supplementary information: Identifying signatures of positive selection in human populations from North Africa

Rocio Caro-Consuegra[1], Marcel Lucas-Sánchez[1], David Comas[1], Elena Bosch[1,2*]

[1]Institut de Biologia Evolutiva (UPF-CSIC), Departament de Medicina i Ciències de la Vida, Universitat Pompeu Fabra, Parc de Recerca Biomèdica de Barcelona, 08003, Barcelona, Spain

[2] Centro de Investigación Biomédica en Red de Salud Mental, Instituto de Salud Carlos III, 28029, Madrid, Spain

*Corresponding author: elena.bosch@upf.edu

This Supplementary Information file contains: Supplementary Figures S1-S4, Supplementary Table captions for Supplementary Tables S1-S11 (in corresponding excel files) and Supplementary Methods.

Supplementary Methods

To understand the observed differentiation of the Tunisian Imazighen populations from Chenini and Sened, we performed the tests described below.

Runs of homozygosity

Runs of homozygosity (ROH) were detected at an individual level in the pruned dataset using PLINK 1.9 with a sliding-window size of 25 SNPs and the remaining parameters set as default. For each population, we computed the per-individual total number, per-individual cumulative length, and per-individual average length of ROH.

Identity-by-descent segments

Identity-by-descent (IBD) segments were detected in the non-pruned dataset with the software IBDseq[1] dividing the detection by chromosome and using default parameters. Following the recommendations of Dai et al. (2020)[2], we filtered out all segments with lengths < 3cM, computed the cumulative IBD sharing between individual pairs by adding all their shared IBD segments and removed all pairs with a cumulative length ≥ 12cM, which are little informative to the general IBD sharing profile. We did not apply any upper filter due to the presence in our dataset of populations with large IBD sharing patterns, which would be hidden if an upper filter were applied. We calculated the median and average cumulative IBD sharing per population (**Supplementary Table S1**).

Effective population size

We used the Linkage Disequilibrium (LD)-based R package NeON[3] to calculate the long-term effective population sizes of the populations in the dataset, i.e., the harmonic mean of the effective population size along past generations. We used each chromosome as a replicate to calculate the mean (percentile 50th of the distribution) and confidence intervals (percentiles 5th and 95th). All parameters were set as default.

Supplementary Figures

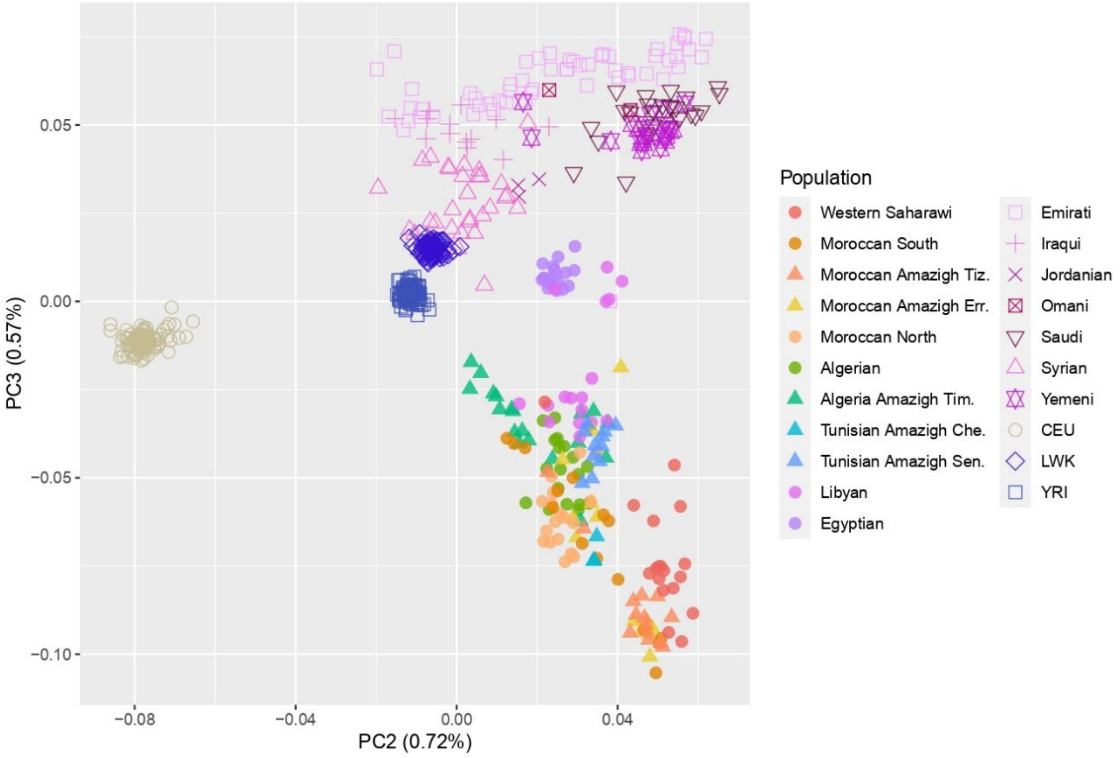

Supplementary Figure S1. PC3 vs PC2 of North African individuals (full coloured symbols) and reference populations (empty symbols) from West and East Africa (YRI and LWK, respectively), Europe (CEU), and the Middle East.

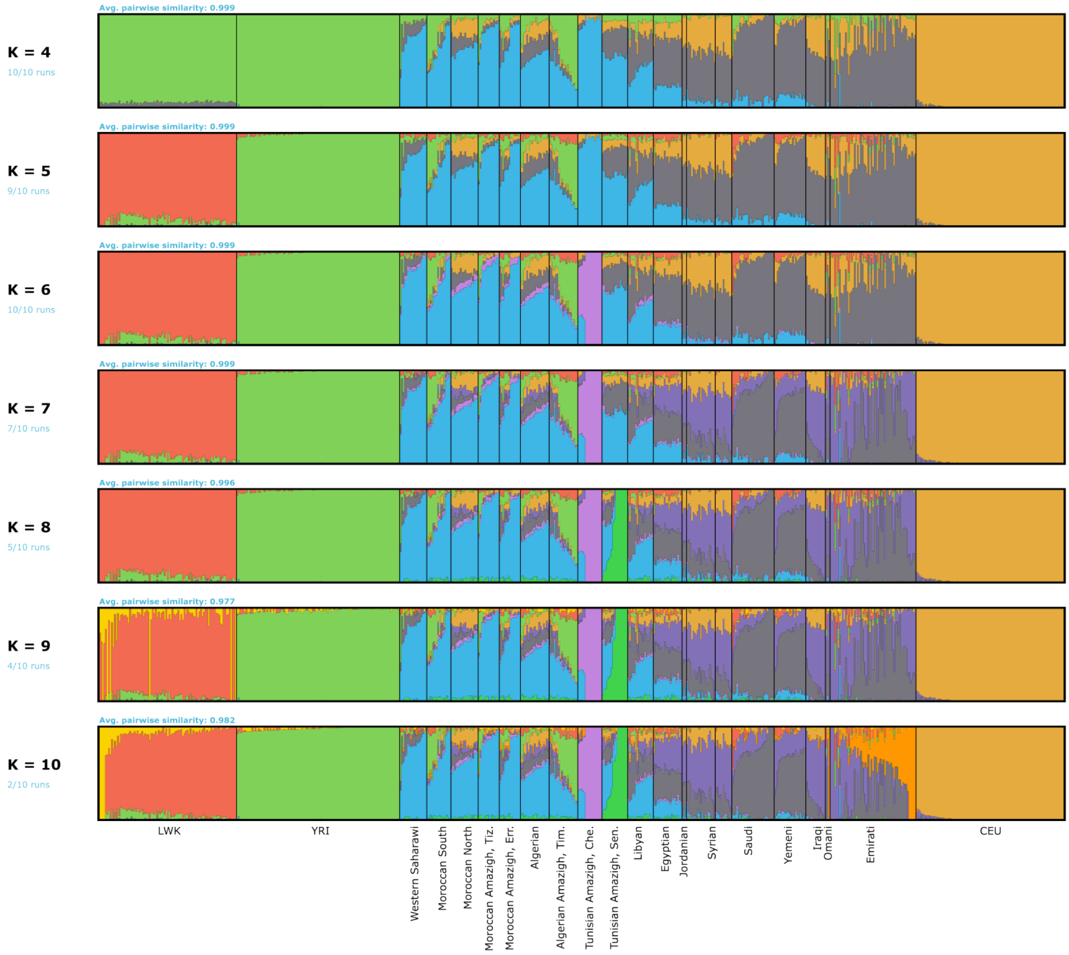

Supplementary Figure S2. ADMIXTURE analysis at K=4-10 with North African individuals and reference populations from West and East Africa (YRI and LWK, respectively), Europe (CEU), and the Middle East.

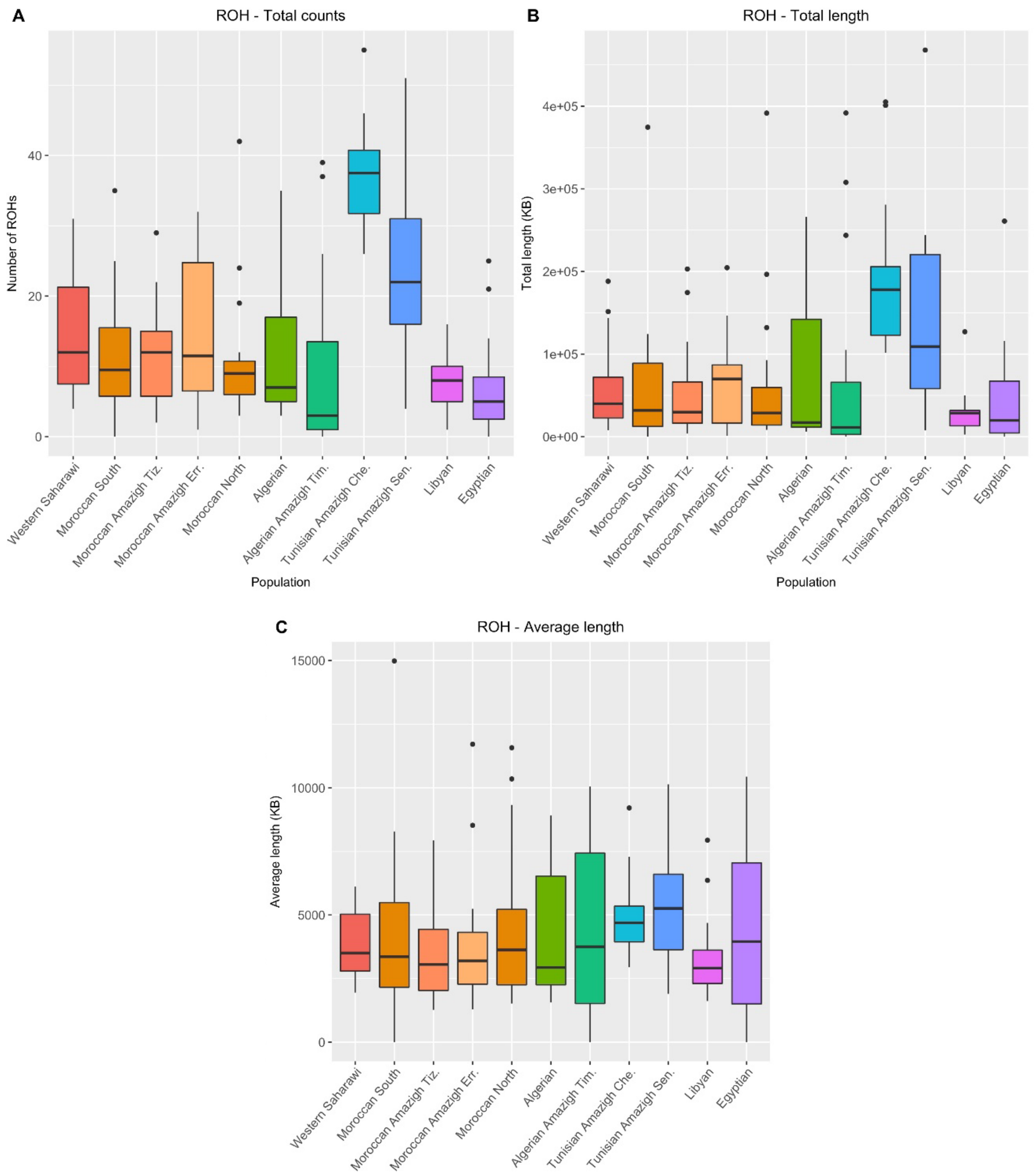

Supplementary Figure S3. Boxplots indicating the distribution of the total counts, total length, and average length of runs of homozygosity (ROH) per individual in each North African population.

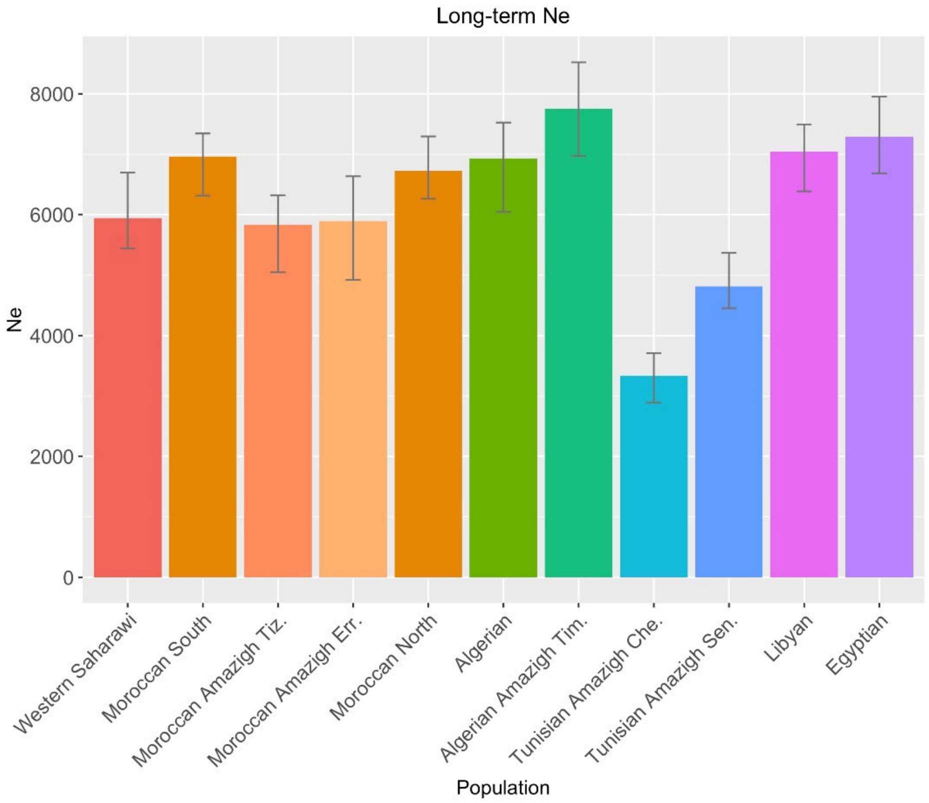

Supplementary Figure S4. Long-term effective population sizes (Ne) of North African populations. Error bars correspond to the 5th and 95th percentiles of the distribution using each chromosome as replicates.

Captions for Supplementary Tables

Table S1. Median cumulative and average cumulative Identity-By-Descent fragments in North African and surrounding populations.

Table S2. Candidate SNPs for selection in North Africa identified within the top 1% F_{ST} and XP-EHH values, when using European (CEU) individuals as the reference population.

Table S3. Candidate SNPs for selection in North Africa identified within the top 1%F_{ST} and XP-EHH values, when using West and East African (WEA) individuals as the reference population.

Table S4. Candidate SNPs for selection in Western North Africa (NAW) identified within the top 0.1% iHS values.

Table S5. Candidate SNPs for selection in Eastern North Africa (NAE) identified within the top 0.1% iHS values.

Table S6. Candidate SNPs for selection identified with a log-likelihood ratio (LLRT) > 15 in the North African ancestry component when using Ohana.

Table S7. Candidate SNPs for post-admixture selection in Western North Africa (NAW) presenting a significant Local Ancestry Deviation (LAD >= 4.42) of the European-like component.

Table S8. List of candidate genes for positive selection exclusively identified in North African (NA) populations. The tests in which each candidate gene was detected are indicated.

Table S9. List of candidate genes for selection identified both in North African (NA) and in the European (CEU) population. The tests of positive selection in which they were detected in each population are indicated.

Table S10. List of candidate genes for selection identified both in North African (NA) and in the West and East African (WEA) populations. The tests of positive selection in which they were detected in each population are indicated.

Table S11. List of candidate genes for positive selection compiled from studies in Southern European populations. The tests of positive selection in which they were detected in each population and references are indicated.

References

1. Browning, B. L. & Browning, S. R. Detecting identity by descent and estimating genotype error rates in sequence data. *Am. J. Hum. Genet.* **93**, 840–851 (2013).

2. Dai, C. L. *et al.* Population Histories of the United States Revealed through Fine-Scale Migration and Haplotype Analysis. *Am. J. Hum. Genet.* **106**, 371–388 (2020).

3. Mezzavilla, M. & Ghirotto, S. Neon: An R package to estimate human effective population size and divergence time from patterns of linkage disequilibrium between SNPS. *J Comput Sci Syst Biol* **8**, 37–44 (2015).
